# Modification of nanodiamonds for fluorescence bioimaging†

**DOI:** 10.1039/d3ra08762j

**Published:** 2024-02-05

**Authors:** Claudia Fryer, Patricia Murray, Haifei Zhang

**Affiliations:** a Department of Chemistry, University of Liverpool Liverpool L69 7ZD UK zhanghf@liverpool.ac.uk; b Department of Women's and Children's Health, Institute of Life Course and Medical Sciences, University of Liverpool Liverpool L69 3GE UK p.a.murray@liverpool.ac.uk

## Abstract

Non-invasive bioimaging is essential in enhancing pre-clinical diagnosis and therapy. Developing efficient imaging probes with high stability, low toxicity, and the potential of offering high resolution images is a very important aspect of developing non-invasive bioimaging techniques. Fluorescent nanodiamonds, which are produced by high energy beam irradiation and high temperature/pressure treatment, have been extensively investigated. In this study, we report the chemical modification of common nanodiamonds (prepared by detonation and high-pressure high-temperature milling) using a stable fluorophore (perylene diimide derivative) *via* carbodiimide coupling. The resulting nanodiamonds show good biocompatibility, cellular uptake and fluorescent imaging potential with mesenchymal stromal cells. This method provides an efficient alternative approach to the preparation and the use of fluorescent nanodiamonds for bioimaging, with the potential benefit of chemically adjusting the structure of perylene diimide for optimized emission/absorbance wavelength.

## Abstract 2

Detonated and milled nanodiamonds are modified with a fluorescent perylene diimide. The resulting nanodiamonds show enhanced fluorescence, good biocompatibility & uptake by mesenchyml stromal cells, and high fluorescence imaging quality.

## Graphical abstract

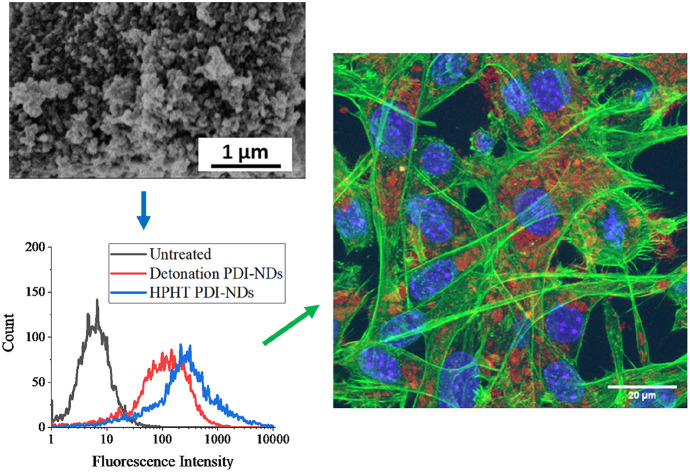

## Introduction

Non-invasive bioimaging techniques can be used to visualize biological events, *e.g.*, monitoring cell growth, migration and differentiation *in vivo*.^1–3^ These techniques are essential for assessing regenerative medicine therapies and for pre-clinical diagnosis and guided therapy.^1–5^ Both molecular probes and nanoparticle probes have been employed to improve imaging contrast and image resolution.^4–6^ Among various imaging techniques, fluorescence imaging and photoacoustic imaging techniques have gained much interest because of their non-invasive potential for intra-operative use, wide availability of nanoprobes, and generally low toxicity with the use of organic nanoprobes.^1,3,6–8^ These organic nanoprobes are usually made of semiconducting or conjugated polymers or alternatively from polyaromatic molecules such as perylene diimides.^3,7,8^ Coordinated polymers and polyphenol-based polymer nanoparticles may also be used as probes for bioimaging but loading or complexation of metal ions onto these polymers is often required.^9,10^

To be used as efficient imaging probes, it is essential to offer high photostability, biocompatibility and low toxicity. In recent years, nanodiamonds (NDs) have emerged as excellent candidates for bioimaging, biosensing and drug delivery.^11–17^ This can be attributed to the excellent properties offered by NDs such as high photostability (nearly quantitative yield), excellent biocompatibility, high chemical stability and great thermal conductivity.^11–13^

NDs were initially studied in the 1960s but have gradually gained increasing interests as a novel type of carbon-based materials.^18^ The diamond structure consists of a dense network of sp^3^ hybridised carbon atoms, each with tetrahedral symmetry, contributing to high chemical inertness. Commercial NDs are usually prepared by high-pressure high-temperature (HPHT) and detonation methods while other methods such as chemical vapour deposition, ball milling and laser ablation have also been investigated.^13,14,19^ The fluorescent properties of NDs usually come from the nitrogen-vacancy (NV) centre, generated from the high energy beam irradiation induced vacancy defects with nitrogen centre available nearby.^15,19^ There are two types of NVs, neutral NV^0^ (emission ∼575 nm) and negatively charged NV^−^ (emission 637 nm), with the NV^−^ commonly employed for bioimaging and biosensing.^12,15,20^ Other atoms, such as Si, may be used as a substitutional atom next to a carbon vacancy (emission ∼ 738 nm) to provide the capacity for dual-colour imaging.^20^

Surface chemical modification of NDs has been extensively investigated, primarily to address colloidal stability, biocompatibility, and interaction with surrounding tissues.^15–17^ These properties, combined with high photostability, lead to wide applications of NDs in biomedicine,^11–17^ including drug delivery and long-term cell tracking.^21^ For example, carboxyl groups on the surface of NDs were modified with poly(ethylene glycol) (PEG) and subsequent physisorption of doxorubicin (DOX), achieving higher drug loading and increased drug efficacy.^22^ For cell tracking, fluorescent NDs with different sizes and concentrations were investigated for cytotoxicity and uptake by mesenchymal stromal cells (MSCs).^23^ Multiple imaging techniques including fluorescence imaging, atomic force microscopy, and 3D soft X-ray tomography were employed to investigate cancer cells with fluorescence NDs.^24^ The long-term imaging potential of NDs was demonstrated by sentinel lymph node mapping, 37 days after injection of NDs in a mouse model.^25^ It was found that after modifying fluorescent NDs with poly(glycerol) and mannose, increased retention and hence enhanced *in vivo* visualization of sentinel lymph nodes was achieved.^26^

However, the widespread use of NDs with NV defects as imaging probes is hampered by the challenges in creating NV centres, as it requires high energy beams and subsequent high-temperature annealing.^13,15^ Therefore, the surface modification of normal NDs (*e.g.*, those directly prepared by detonation and high-pressure high-temperature synthesis) with fluorophores can offer an effective alternative route.^27^ This can be achieved by either covalent modification or non-covalent modification (*i.e.*, physical adsorption).^28,29^ For instance, poly-l-lysine was used to animate the ND surface with subsequent immobilisation of Cytochrome C.^30^ Octadecylamine was covalently attached to 5 nm NDs, exhibiting peak excitation and emission wavelengths of 410 and 450 nm, respectively.^27^

Because the photostability of fluorescent NDs is highly desirable, it is very important to select stable fluorophores to modify the NDs. Perylene diimide (PDI) derivatives have emerged as excellent candidates for fluorescence emission and near infrared absorption with high quantum yield and great photostability.^31,32^ Various chemistry strategies can be applied to vary emission/absorption wavelength and surface functionality.^33,34^ We reported before the use of PDI nanoparticles for *in vitro* and *in vivo* imaging of MSCs by fluorescence imaging and photoacoustic imaging.^3^ It is thus a good step forward to modify the NDs with PDI molecules in order to achieve the generation of fluorescent NDs with combined advantages of NDs and PDIs.

Herein, we report the surface modification of NDs using a fluorescent PDI. These PDI-NDs are characterised and evaluated as nanoprobes for fluorescence imaging. MSCc are labelled with the resulting PDI-NDs in order to assess the cytotoxicity and cellular uptake of the probes. The novelty of this work lies in the capability of chemically modifying NDs with stable PDI fluorescent dyes and its valid use for fluorescence imaging. It offers the potential for long-term cell tracking in regenerative medicine.

## Results

Two types of NDs were used, amine-functionalised NDs prepared by detonation NDs (5–10 nm) and milled HPHT NDs (∼100 nm). The effect of size and surface functionality of the NDs on surface modification, cell toxicity, cell uptake and fluorescent imaging by confocal laser microscopy, were investigated. Both types of NDs were modified by covalently attaching the PDI molecules to the surface of the NDs.

### NDs and modification with PDI

**Fig. 1:**
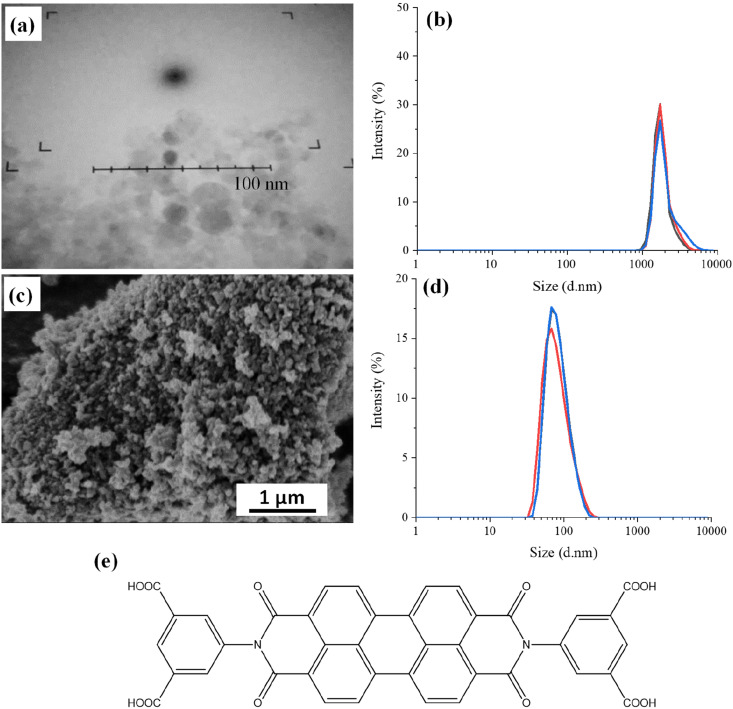
The NDs and PDI used in this study: (a) & (b) TEM image and the DLS profile of detonation NDs (5–10 nm). (c) & (d) SEM image and DLS profile of the HPHT NDs (∼100 nm). (e) Molecular structure of the PDI.

The detonation NDs with amino groups on the surface have particle sizes typically in the range of 5–10 nm, as shown by the Transmission Electron Microscopy (TEM) image (Fig. 1a). It was difficult to image because these nanoparticles were prone to aggregation and the contrast between NDs and the support carbon film on the TEM grid was low. Dynamic Light Scattering (DLS) measurements were carried out to determine the hydrodynamic diameter of the NDs in aqueous suspension. Fig. 1b shows the volume particle size distribution as this considers the spherical shape of the nanoparticles and Mie scattering. The particle size of the detonation NDs is shown at 1811 ± 468 nm with a polydispersity index of 1.52, suggesting the nanoparticles are highly aggregated to a much larger size. The presence of amino groups on the surface of the NDs was confirmed by the positive zeta potential (+23.6 ± 9.82 mV).

For the milled HPHT NDs, Fig. 1c shows approximate sizes of around 100 nm. These NDs were diluted and sonicated in an ultrasound bath for a DLS analysis. The measured hydrodynamic diameter of the particles was 100 ± 30 nm with a low particle size distribution of 0.065 (Fig. 1d). Hence, there is limited aggregation in aqueous suspension for these milled larger NDs, compared to the smaller detonation NDs. The zeta potential was found to be −33.4 ± 6.35 mV, which is consistent with milled NDs, which usually exhibit hydrogen/hydroxyl terminating groups on the surface.^23^

The detonation NDs with surface amine groups were directly modified with PDI, *i.e.*, without any further treatment. The molecular structure of the PDI is shown in Fig. 1e. This PDI has poor solubility in common solvents, including neutral water, and hence basic water was used for the reaction. PDI was first dissolved in basic water (0.06 mM NaOH, pH = 8.2) and 1-ethyl-3-(3-dimethylaminopropyl)carbodiimide hydrochloride (EDC) and *N*-hydroxysulfosuccinimide sodium salt (sulfo-NHS) were subsequently added. After adding the detonation NDs and completing the reaction at room temperature overnight, the suspension was centrifuged and resuspended in deionized water to yield a cloudy pink suspension. This pink suspension was centrifuged again. UV-visible (UV-vis) spectroscopic analysis was carried out with the supernatant phase, which showed no absorption in the visible spectrum. The UV-vis spectrum of the PDI solution is given below in the section of optical properties of PDI-modified NDs. This suggested that the PDI was attached to the NDs, rather than dissolved in the basic water.

**Fig. 2:**
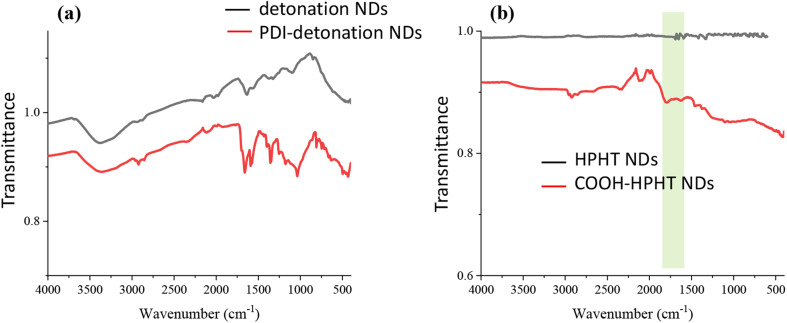
FTIR spectra of the as-used NDs and modified NDs. (a) The detonation NDs and after modification with the PDI. (b) The HPHT NDs and after modification with carboxylic acid surface groups. The light green bar indicates the presence of –COOH group.

The zeta potential of PDI-NDs significantly decreased to −19.2 ± 4.87 mV, further suggesting the attachment of PDI to the detonation NDs. The reaction was also confirmed using FTIR spectroscopy. Before the modification with PDI, the FTIR spectrum in Fig. 2a shows the broad stretch at 3300–3500 cm^−1^ associated with free amine groups on ND surface and the stretch at 1630 cm^−1^ indicative of N–H bending. After the modification, new stretches are observed around 1580 and 1350 cm^−1^ for PDI-NDs (Fig. 2a). The stretch at 1580 cm^−1^ can be attributed to the presence of aromatic C=C and the stretches at 1350 cm^−1^ the C=O of free carboxylic acid groups, thus suggesting the presence of the PDI derivative.^35^ Furthermore, the stretch at 1650 cm^−1^ is characteristic of C=O amide bond.^11^ The broad stretch at 3300–3500 cm^−1^ is associated with free amine groups on ND surface; this can be seen in the unmodified NDs and the PDI-NDs, suggesting that not all the amine groups had reacted.

The PDI-ND suspensions were prone to aggregation, partly due to the high concentration of the suspension (1 mg ml^−1^) and hence diluted suspensions were used for further characterisation. A concentration of 0.1 mg ml^−1^ was used for DLS (after sonication for 10 minutes using an ultrasonic bath) following guidance from the manufacturer. However, the aggregation issue remained. The particle size of PDI-NDs was similar to the unmodified NDs at 1793 ± 227 nm and the polydispersity index was 0.363 (Fig. S1†).

The HPHT NDs were first treated with concentrated H_2_SO_4_/HNO_3_ to introduce COOH groups to the ND surface, before the modification with PDI. This was confirmed by measuring the zeta potential of the modified NDs. For the HPHT NDs functionalized with –COOH groups, a zeta potential of −57.6 ± 12.1 mV was measured, which is significantly more negative than before the modification (−33.4 ± 6.35 mV). The broad stretch from 1650–1850 cm^−1^ by FTIR analysis in Fig. 2b suggests that carboxylic acid groups were introduced to the ND surface.

PDI was attached to the surface of the COOH-HPHT NDs using the same protocol as for the detonation NDs with EDC/sulfo-NHS, only this time to form an anhydride bond rather than amide. The suspension of PDI-HPHT NDs was cloudy pink in colour compared to the grey colour of the NDs before PDI modification. Furthermore, after centrifugation, the supernatant was colourless suggesting PDI had been successful attached. The particle size distribution remained consistent with the unreacted COOH-NDs at 106 ± 41 nm (Fig. S2†) and the polydispersity index was 0.136. The zeta potential was −54.3 ± 8.23 nm which was similar to the value before the PDI modification. For PDI-NDs prepared with detonation NDs with amine surface functionality, there was a significant decrease in zeta potential from +23.6 ± 9.82 mV to −19.2 ± 4.87 mV. However, in this case, as the COOH-NDs already had a negative zeta potential, it is not unusual that the zeta potential did not change much with the addition of PDI. The FTIR spectrum of the larger PDI-NDs (Fig. S3†) was similar to that of PDI-NDs prepared from detonation NDs. The stretch at 1580 cm^−1^ is consistent with C=C and the stretches at 1350 cm^−1^ and 1650 cm^−1^ can be associated with C=O of PDI.

### Optical properties of PDI-modified NDs

**Fig. 3:**
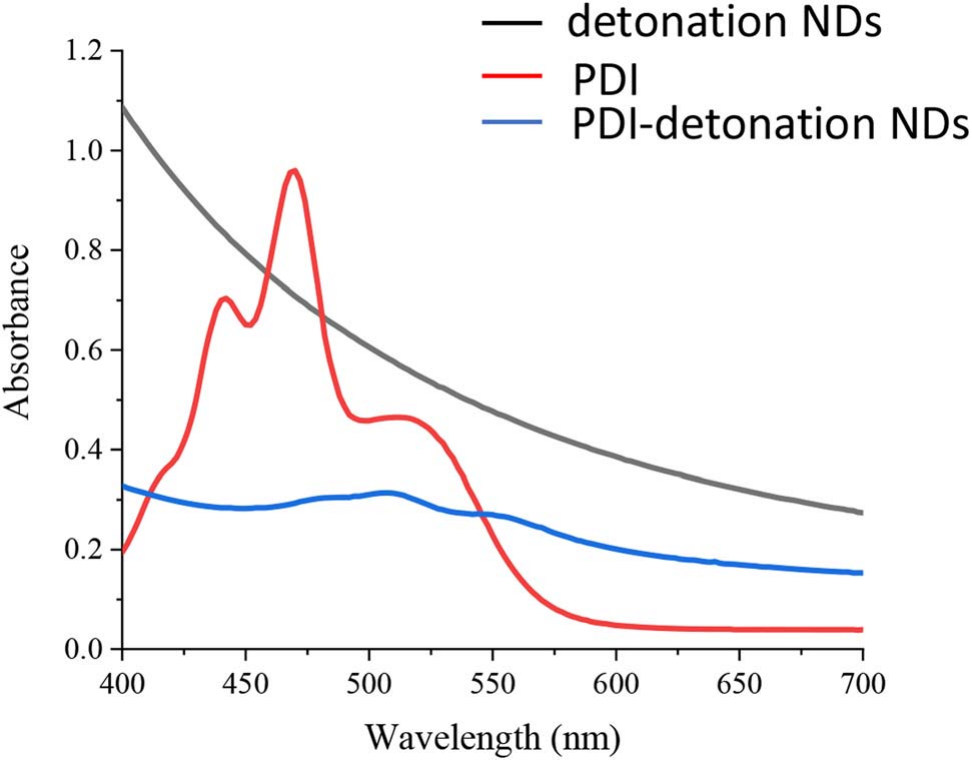
UV-vis spectra of PDI (0.1 mg ml^−1^ in 0.06 mM NaOH), detonation NDs and the PDI-modified detonation NDs (neutral water, 0.1 mg ml^−1^).

The optical properties of the PDI-modified NDs were assessed using UV-vis spectroscopy and fluorescence spectrometry. The UV-vis spectrum of the PDI shows the peaks at 440, 470 and 510 nm (Fig. 3). When the PDI-modified detonation NDs were analysed, there was very high background signal due to the inherent light scattering of the NDs; this is consistent with results found in the literature.^30^ The peaks attributed to PDI in the modified NDs were also significantly lower (Fig. 3). This is likely due to the low amount of PDI in PDI-NDs compared to equivalent mass concentration of pure PDI. Higher concentrations of PDI-NDs could increase peak intensity but at the expense of increased light scattering. UV-vis spectroscopy of the PDI-HPHT NDs was similar to PDI-detonation NDs in that there was a large background signal due to the light scattering of the NDs (Fig. S4†). A slight peak could be observed around 460 nm which is similar to that of PDI alone.

**Fig. 4:**
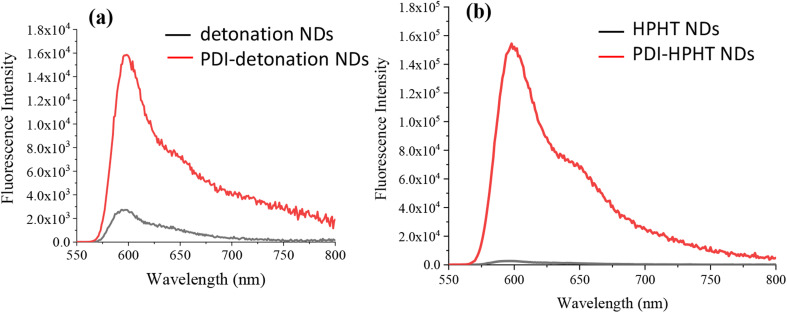
Fluorescence emission spectra (*λ*_ex_ = 500 nm) for (a) detonation NDs and the PDI-modified detonation NDs. (b) HPHT NDs and the PDI-modified HPHT NDs. Suspensions with a solid concentration of 0.1 mg ml^−1^ in water were measured. An emission filter was used to block emission below 590 nm to counteract the effect of light scattering.

The fluorescence property of the PDI-NDs was assessed and compared with that of the free PDI dye and a sample of just NDs. The emission spectra for pure PDI showed high emission intensity at 600 nm (Fig. S5†). When detonation NDs and PDI-detonation NDs were analysed at the same excitation wavelength, a 590 nm emission filter was needed to counteract the light scattering effects. As shown in Fig. 4a, the emission spectra of detonation NDs exhibits very low fluorescence, mostly associated with background signal and effects from the emission filter; however, for PDI-detonation NDs the significant characteristic peak for PDI emission is displayed at 600 nm. Similar enhanced emission was also observed for PDI-HPHT NDs (Fig. 4b).

### Evaluation of PDI-modified NDs as fluorescent probes

**Fig. 5:**
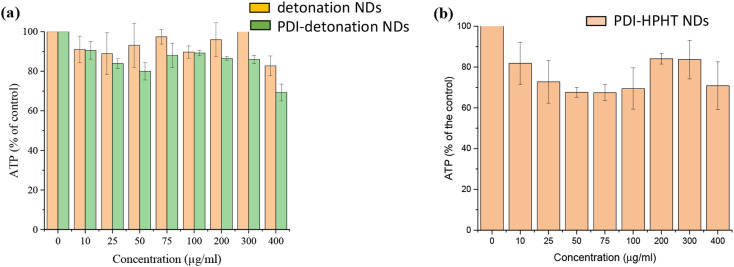
The effect of PDI-modified NDs on ATP production in MSCs. (a) Unmodified detonation NDs and PDI-modified NDs. (b) PDI-modified HPHT NDs. Cells were seeded at a density of 2 × 10^5^ cells per well (96-well plate) and incubated for 24 hours before dosing with NDs for a further 24 hours. The error bars represent standard deviation of biological repeats (*n* = 3).

It was essential to confirm the biocompatibility of the NDs and whether PDI conjugation affected their biocompatibility. The biocompatibility was assessed by the CellTiter-Glo® luminescent cell viability assay. The assay involves cellular ATP (adenosine triphosphate) released from lysed cells which react with luciferin/luciferase, leading to light emission at 562 nm. Cell viability was measured as the amount of ATP released from treated cells as a percentage of the ATP released from the control, as shown in Fig. 5a. Both detonation NDs and PDI-detonation NDs have limited effect on the viability of MSCs up to concentrations of 300 μg ml^−1^. The cell viability was slightly lower for PDI-detonation NDs; however, it remained above 80% up to a ND concentration of 300 μg ml^−1^.

The cytotoxicity of the HPHT PDI-NDs was also assessed (Fig. 5b). These appeared to have a greater cytotoxic effect on MSCs compared to the PDI-detonation NDs. About 70% or higher viability was observed for all the concentrations of HPHT PDI-NDs suspensions tested. While the viability decreased to ∼70% at a concentration of 50 μg ml^−1^, no further decrease was observed as the concentration was increased to 400 μg ml^−1^, with viability at all concentrations from 25–400 μg ml^−1^ remaining between 70–80%.

**Fig. 6:**
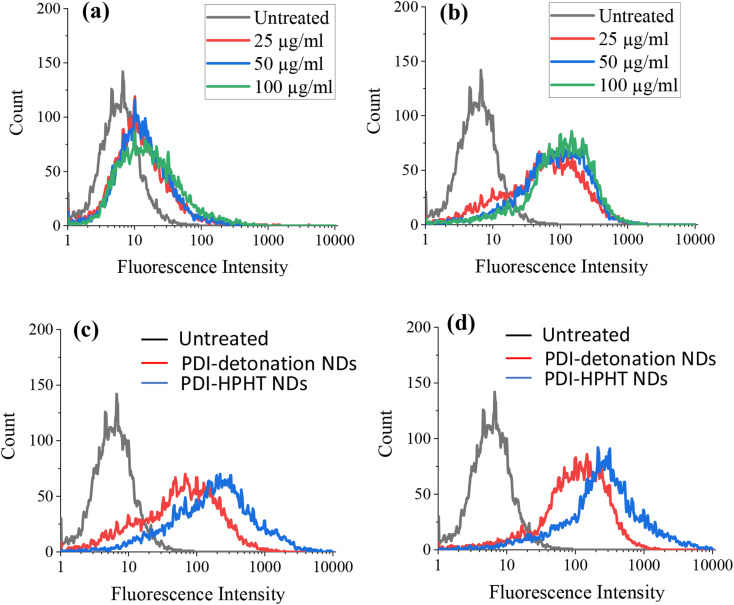
Live cell flow cytometry of MSCs in PBS labelled with various concentrations of (a) unmodified detonation NDs and (b) PDI-modified detonation NDs for 24 hours. The fluorescence intensity was then compared between the MSCs labelled with PDI-detonation NDs and PDI-HPHT NDs at the ND concentration of (c) 25 μg ml^−1^ and (d) 100 μg ml^−1^. Fluorescence intensity was measured using FL3 filter (670 LP), where 10 000 events were counted.

It was possible that the low cytotoxicity could be due to limited cellular uptake, particularly for the large ND aggregates. Therefore, the uptake of PDI-modified NDs by the MSCs was assessed by flow cytometry. For flow cytometry, MSCs were seeded at a density of 2 × 10^6^ cells per well and incubated for 24 hours ahead of dosing with PDI-modified NDs. The media was aspirated and replaced with fresh media containing NDs at concentrations of 25, 50 or 100 μg ml^−1^. Both unmodified and PDI-modified NDs were investigated. The MSCs that were not treated with NDs were used as a control. Flow cytometry was then carried out on live cell suspensions in PBS (10 000 cells were counted for each sample), as shown in Fig. 6.

An increase in fluorescence intensity is observed for the MSCs treated with both unmodified (Fig. 6a) and PDI-modified detonation NDs (Fig. 6b). The increased fluorescence intensity is more significant at all tested PDI-ND concentrations, as clearly demonstrated in Fig. S6.† A similar increase of fluorescence intensity was also demonstrated when the MSCs were treated with PDI-modified HPHT NDs (Fig. S7†). The fluorescence intensity of both sets of PDI-NDs (detonation and HPHT) was compared and greater fluorescence was seen for those MSCs treated with PDI-HPHT NDs (Fig. 6c and d). This suggests that there is greater cellular uptake of these particles, possibly due to reduced aggregation.

**Fig. 7:**
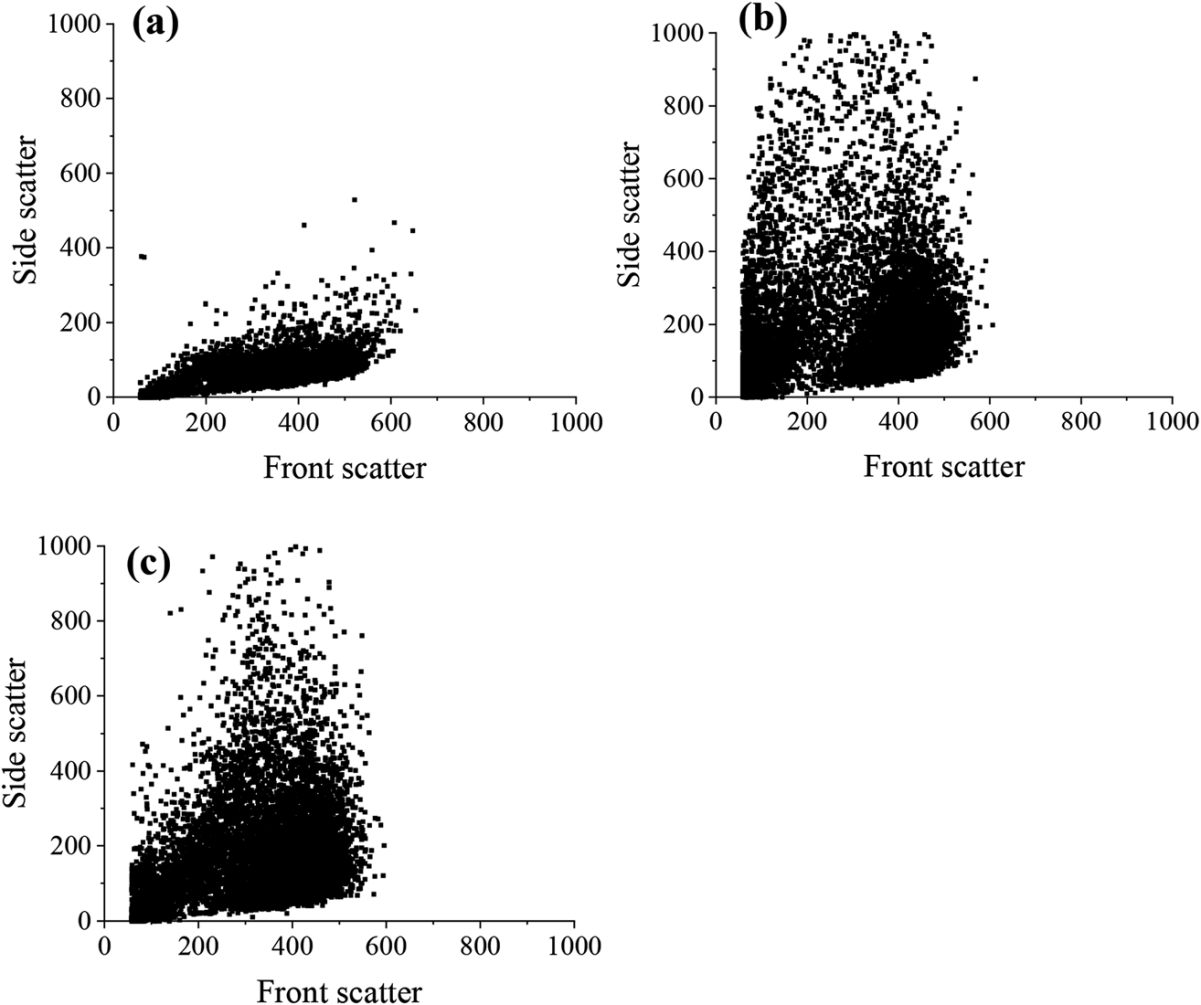
Flow cytometry dot plots showing changes in front scatter and side scatter for (a) untreated MSCs and MSCs treated with (b) unmodified detonation NDs and (c) PDI-modified detonation NDs, respectively, at a concentration of 25 μg ml^−1^.

The flow cytometry data was also represented as dot plots, showing the change in the side and front scatter of cells for untreated and ND-treated MSCs (Fig. 7). The front scatter indicates the size of the cell and side scatter is related to the cell complexity or granularity.^36^ There was an increase in the side scatter for MSCs labelled with both PDI-detonation NDs and unmodified detonated NDs (Fig. 7a–c), which suggests a change in cell granularity. A similar dot plot was obtained for PDI-modified HPHT NDs (Fig. S8†). This confirms that the change in fluorescence intensity is due to the ND uptake.

### Visualising cellular uptake with confocal microscopy

Flow cytometry provided information on the proportion of fluorescent cells and degree of intensity per cell. However, confocal microscopy was required to further assess the uptake of PDI-NDs into the MSCs, as it was possible that these particles could be associated with the surface rather than inside the cells. With confocal microscopy, various planes can be imaged through the cell to confirm the internalisation and distribution of particles.

Confocal microscopy was carried out on fixed samples after 24 hour dosing with PDI-modified NDs. Multiple images were taken at different focal planes and combined to form Z-stack images with a greater depth of field. The obtained images could provide evidence of whether the NDs were inside cells and not just associated with the cell surface.

**Fig. 8:**
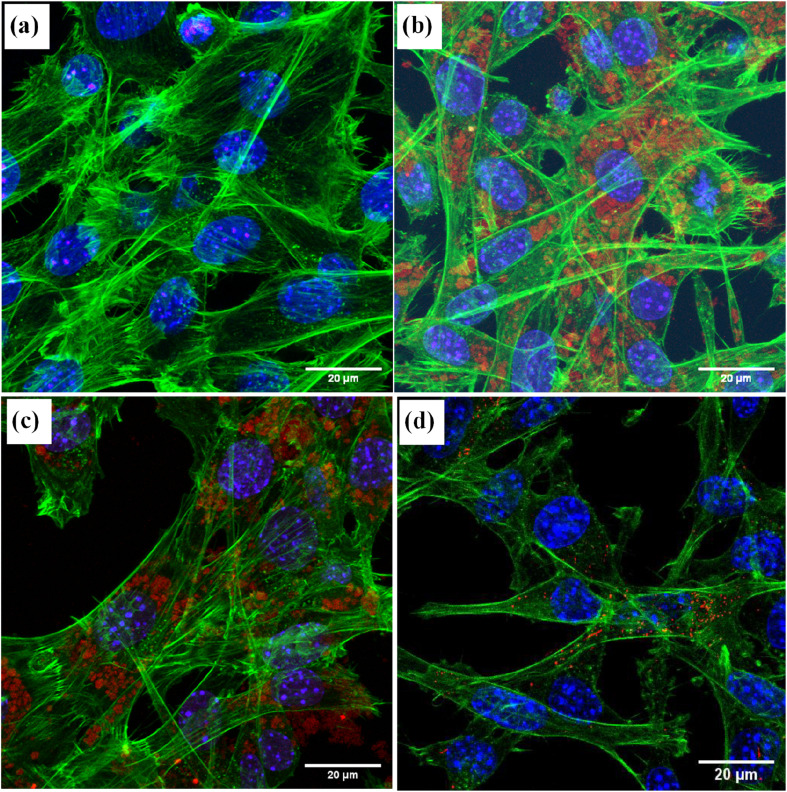
Representative confocal fluorescence microscopic images of MSCs treated with (a) unmodified detonation NDs, (b) PDI-modified detonation NDs, (c) PDI-modified HPHT NDs, and (d) PDI nanoparticles (as a control). Cells were dosed at a concentration of 50 μg ml^−1^ for 24 hours and then were fixed and stained with DAPI (blue nuclei) and AlexaFluor ® 488 phalloidin (green, actin cytoskeleton). PDI-modified NDs and PDI nanoparticles can be seen in red within the MSCs.

Fig. 8 shows the images of the MSCs treated with different types of NDs. Unmodified detonation NDs could be hardly detected (Fig. 8a) while the PDI-detonations NDs could be clearly visualised (red spots) (Fig. 8b). It is noticeable that the red spots generated from the PDI-detonation NDs are located separately from the blue parts (stained nuclei) and the green parts (stained actin cytoskeleton). This pattern suggests the NDs within the cells is likely to be in an endosomal location.^37,38^ It appears that the majority of cells were labelled, which agrees with the flow cytometry data. Similarly, the PDI-HPHT NDs could be visualised within MSCs as aggregates (Fig. 8c). The degree of cell labelling appears to be similar to the detonation PDI-NDs, where the majority of cells were labelled. As a control, the image from the MSCs treated with PDI nanoparticles (*i.e.*, no NDs involved) is shown in Fig. 8d. Because PDI is only soluble in basic water (*i.e.*, insoluble under physiological conditions), PDI nanoparticles (∼150 nm, prepared by nanoprecipitation – dropwise addition of 1 mg ml^−1^ PDI–acetone solution into aqueous polyvinyl alcohol solution) had to be produced for use in bioimaging. As such, directly conjugating the PDI to NDs circumvents the process of preparing PDI nanoparticles. Because PDI is covalently conjugated to the ND *via* the formation of amide bond (*i.e.*, not physical adsorption), it is unlikely that PDI would be cleaved off the NDs while inside the MSCs. This indicates that the fluorescence imaging capability of modified NDs indeed resulted from the PDI modification.

## Discussion

Two ND samples, *i.e.*, detonation NDs (5–10 nm) and HPHT NDs (50 nm) were functionalised with PDI, an organic fluorophore. Before modification, the NDs were first characterised with SEM and DLS to investigate the particle size. Detonation NDs were found to be their expected particle size of 5–10 nm by TEM. However, when analysing the aqueous ND suspension with DLS, a particle size of just under 2000 nm and a high polydispersity index of 1.52 were measured (Fig. 1). This suggests that the small detonation NDs exist mainly as aggregates in aqueous medium. The HPHT NDs used in this study were found to be ∼100 nm with DLS and had a low polydispersity index of 0.065 (Fig. 1). This indicates that the larger HPHT NDs are more likely not to aggregate in aqueous suspension.

FTIR spectroscopy was used to confirm the presence of amino groups and carboxylic acid groups on the detonation NDs and COOH-functionalized HPHT NDs, respectively (Fig. 2). Broad absorption was observed in the fingerprint region up to ∼1800 cm^−1^ which is characteristic of NDs, coming from the C=O stretches and other overlapping peaks.^11^ The absorption can be explained by overlapping stretches due to the many bonding stretches present in the NDs structure. The amino and carboxylic acid groups were utilised for carbodiimide coupling to the fluorescent dye, PDI.

The amino groups on the surface of the detonation NDs were employed for carbodiimide coupling to the free carboxylic acid groups in the PDI derivative. EDC and sulfo-NHS were employed to improve the efficiency of the coupling reaction, particularly because of their good solubility in water which is ideal for the biomedical applications of the probes.^39^

The coupling experiment was carried out in basic water (0.06 mM NaOH) due to the limited solubility of the PDI derivative in neutral water.^40^ Although neutral pH is optimal for EDC, the basic pH was necessary for solubilising the PDI derivative. Sulfo-NHS was included to improve reaction efficiency by reducing the formation of the stable *N*-acylurea side product. The resultant product after the coupling reaction was a cloudy, pink PDI-ND suspension. The attachment of PDI was confirmed with FTIR where stretches could be seen which corresponds to the carboxylic acid groups and aromatic structure of PDI, as well as a new peak associated with amide bond (1650 cm^−1^) (Fig. 2). There were also stretches associated with free amine groups however the zeta potential significantly decreased from +23.6 ± 9.82 mV for the unmodified NDs to −19.2 ± 4.87 mV, indicating the successful attachment of PDI onto the detonation NDs. The PDI-detonation NDs still appeared to aggregate as the particle size remained ∼2000 nm by DLS.

UV-vis spectrometry and fluorescence analysis further suggested the presence of PDI, as small peaks could be seen which correspond to the wavelengths associated with PDI alone (Fig. 3). It should be noted that the absorbance/fluorescence data is not ideal due to the high refractive index (RI) of NDs resulting in significant scattering.^19,30^ Peaks at 470 and 500 nm can be seen in the UV-vis spectrum and an emission peak at 600 nm were observed after excitation at 500 nm (Fig. 4). These wavelengths are consistent with that of free PDI. Hence, the probes were taken forward for *in vitro* cell studies, as the optical properties appeared promising.

The murine MSC cell line was used for *in vitro* studies, where cells were labelled by incubation with media containing NDs for 24 hours. Both detonation NDs and PDI-detonation NDs had a limited cytotoxic effect in MSCs up to concentrations of 300 μg ml^−1^ (Fig. 5). This is consistent with the literature where the high biocompatibility of NDs in various cell lines has been demonstrated.^11,19,23^ The conjugation of PDI did not affect cell viability significantly, which suggests the centrifuge washes were effective in removing EDC/sulfo-NHS. The concentration of 300 μg ml^−1^ for cytotoxicity is high, especially compared to polymer-stabilised PDI nanoparticles.^3^

Flow cytometry and confocal microscopy were then used to assess cellular uptake as it was possible that the low cytotoxic effect could be attributed to limited uptake, particularly as DLS suggested NDs existed predominantly as large aggregates. MSCs were labelled with detonation NDs and PDI-detonation NDs for 24 hours and flow cytometry was carried out on live cell suspensions in PBS (Fig. 6). The MSCs labelled with PDI-detonation NDs exhibited increased fluorescence compared to untreated MSCs. Cells labelled with unmodified detonation NDs showed a slight increase in fluorescence but it was not as noteworthy as with the PDI-detonation NDs. Both PDI-detonation NDs and unmodified detonation NDs showed very little change in fluorescence intensity with increasing dosing concentrations. And the width of the peaks did not change noticeably. This suggests that increasing the dosing concentration did not affect the amount of NDs taken up per cell under the investigative conditions.

For the flow cytometry experiments, there was a change in the laser side scatter between the untreated cells and both PDI-detonation NDs and unmodified detonation NDs (Fig. 7). Side scatter is related to cell granularity, which is an indication of cellular uptake thus suggesting that the cells had taken up both unmodified detonation NDs and PDI-detonation NDs. It is also worth noting that the high RI of NDs may also affect laser scattering. The side scatter was similar for PDI-detonation NDs and unmodified detonation NDs, which suggests that there was a similar amount of cellular uptake for both sets of particles. The difference in fluorescence intensity but similar side scatter suggests that PDI-detonation NDs have greater fluorescence at 670 nm than unmodified detonation NDs.

Confocal laser microscopy was then carried out to visualise the PDI-detonation NDs within the cells (Fig. 8). PDI-detonation NDs could be observed in the majority of cells, which is consistent with the flow cytometry data. Unmodified detonation NDs were not detected by confocal laser microscopy, as a result of very low fluorescent intensity (Fig. 6a).

Although the detonation NDs had low cytotoxicity, the aggregation was not ideal. Hence, larger HPHT NDs with hydrogen/hydroxyl terminating groups, prepared by a milling process, were also investigated. To achieve the PDI functionalization, carboxylic acid groups were required to be introduced to the HPHT ND surface using an acid treatment, which was confirmed by the highly negative zeta potential (−57.6 ± 12.1 mV) and FTIR measurement (Fig. 2b and S3†). Similarly, the PDI-HPHT NDs were prepared and characterized, which showed satisfactory cytotoxicity (Fig. 5b) and similar or moderately better fluorescent properties (Fig. 4, 6 and S8†). The uptake of PDI-HPHT NDs in MSCs was subsequently investigated using confocal laser microscopy. A good imaging performance was demonstrated, similarly to the PDI-detonation NDs (Fig. 8). This suggests this PDI-modification approach may be applicable for different types of NDs. Considering the potential of chemically varying the PDI molecular structure and hence the fluorescent/near infrared properties,^32–34^ this can provide a highly efficient yet versatile platform for bioimaging.

## Conclusion

Two types of NDs with different sizes, *i.e.*, detonation NDs (5–10 nm) and HPHT NDs (∼100 nm), have been functionalized with PDI and then evaluated as nanoprobes for fluorescence imaging. Detonation NDs with amino groups on the surface were directly functionalized *via* carbodiimide coupling while the HPHT NDs were first treated with acids to introduce –COOH groups. The PDI-functionalized NDs showed good biocompatibility and fluorescent properties, as evidenced by cytotoxicity and flow cytometry assays. The uptake of these functionalized fluorescent NDs by MSCs allowed for fluorescent imaging by confocal laser spectroscopy. This provides an efficient and alternative route to the NV^−^ NDs for non-invasive bioimaging. The advancement demonstrated in this work shows it is viable to chemically functionalize NDs with fluorescent PDIs for bioimaging. This advancement can be further explored by the capability of synthesizing PDIs with multiple fluorescence emission wavelengths and the near infra-red absorbance, offering the potentials in multi-modal imaging.

## Experimental methods

### Reagents

All reagents were used as received from the manufacturer. Sodium hydroxide (≥97%), *N*-(3-dimethylaminopropyl)-*N*′-ethylcarbodiimide hydrochloride (EDC) and *N*-hydroxysulfosuccinimide sodium salt (sulfo-NHS) were purchased from Sigma Aldrich. Detonation NDs (5–10 nm) and HPHT NDs (50 nm) were provided by Element Six Ltd. *N*,*N*′-Di(3,5-dicarboxyphenyl)perylene-3,4,9,10-tetracarboxylic diimide (PDI) was synthesized as reported previously.^40^

### Surface modification of HPHT NDs

Carboxylic acid functional groups were introduced to the surface of HPHT NDs using a reported protocol in the literature.^41^ Briefly, 50 nm NDs (2.0 g) were dispersed in 80 ml acid solution (3 : 1 v/v, 69% H_2_SO_4_/37% HNO_3_) and heated to reflux for 24 h. Once cooled, the mixture was slowly added to distilled water and centrifuged (4000 rpm). Particles were washed three times by centrifugation and consequently dried under vacuum for 24 h.

### Conjugation of PDI to detonation and HPHT NDs

The attachment of PDI to the COOH-functionalized NDs was performed using similar procedures as reported in the literature.^42,43^ Typically, PDI (7 mg, 0.01 mmol) was dissolved in 10 ml of basic water (0.06 mM NaOH, pH = 8.2). EDC (26.7 mg, 0.1 mmol) and sulfo-NHS (30.2 mg, 0.1 mmol) were added to 2 ml of the PDI solution. The mixture was stirred at room temperature for 30 minutes before the addition of either detonation or HPHT NDs (2 ml, 0.7 wt%). The ND suspension was stirred overnight and subsequently separated and washed by centrifugation using deionized water.

### Characterisation methods

A Malvern Nano Zetasizer was used to measure the hydrodynamic diameter and zeta potential of NDs in the suspension. Samples were diluted with water (0.1 mg ml^−1^) and sonicated for 10 minutes prior to the analysis. Images of the dry NDs were obtained with a Hitachi S4800 Scanning Electron Microscope. A Vertex 70 Fourier Transform Infrared (FTIR) Spectrometer was used to examine the functionalization of NDs. A μ-Quant Microplate Reader and a Fluorescence Lifetime Spectrometer (FLS) 1000 (Edinburgh Instruments) were employed to assess the light absorption and emission properties of the ND suspension. For the fluorescence measurements, samples were prepared at a concentration of 0.1 mg ml^−1^ and analysed using a front-face sample holder, 590 nm emission filter and excitation/emission bandwidths of 0.8–3 nm. Excitation wavelengths of 500 nm was used and emission was measured in the range of 550–800 nm with a 1 nm step and 0.2 s dwell time.

### Cell culturing

The murine mesenchymal stromal cell line D1 (ATCC) modified to express firefly luciferase was used for the cell studies.^44^ The MSCs were cultured in 6 cm or 10 cm tissue culture dishes (Greiner CELLSTAR®) in high glucose Dulbecco's Modified Eagle Medium (DMEM, Sigma Aldrich) containing 10% fetal bovine serum (FBS, Sigma Aldrich), 1% non-essential amino acids (Sigma Aldrich) and 2 mM l-glutamine (Gibco). Cells were incubated at 37 °C and 5% v/v CO_2_ and passaged with 1% trypsin/EDTA (Sigma Aldrich) when they reached 90% confluence.

### Cell viability assay

CellTiter-Glo® Luminescent Cell Viability Assay (Promega) was utilized. The MSCs were added in triplicate at a density of 2 × 10^5^ cells per well in a 96-well plate and were allowed to grow for 24 h. The medium in the well was then replaced with 200 μl fresh medium containing various concentrations of aqueous ND suspension (0–400 μg ml^−1^). Prior to the assay, the MSCs were incubated for a further 24 h and the cells were washed to remove any excess NDs. A μ–Quant Microplate reader was used to measure the luminescence of the MSCs.

### Flow cytometry analysis

The cellular uptake and fluorescence properties of the NDs were examined by flow cytometry. MSCs were added into a 24-well plate at a density of 2 × 10^6^ cells per well and were allowed to incubate for 24 h. The cell medium was then removed and replaced with fresh medium containing NDs of different concentrations (0–100 μg ml^−1^). After incubating for a further 24 h, the MSCs were resuspended in PBS (1×), which was maintained at low temperature on ice. Flow cytometry analysis was subsequently performed using a BD FACSCalibur™ (BD Biosciences) with a 488 nm laser wavelength and a long pass FL3 filter (670 nm). During the measurement, threshold values for side scatter (SSC), front scatter (FSC) and FL3 were set based on the control sample where the MSCs were not labelled with the NDs. For each measurement, 10 000 events were counted and the data were collected and analysed with Flowing Software 2.5.1 (Turku Bioscience Centre).

### Confocal laser microscopic imaging

In order to confirm the uptake and fluorescence property of the PDI-modified NDs in the MSCs, confocal laser microscopy was used to inspect the samples. MSCs were placed in 8-well chamber slides (Ibidi μ-Slide 8-well) at a density of 2 × 10^5^ cells per well. The MSCs were co-cultured with NDs in the medium with a concentration of 50 or 100 μg ml^−1^ for 24 h. Subsequently, the cells were fixed with 4% paraformaldehyde, permeabilised with Triton X100 (0.1% v/v), and stained with 4′,6-diamidino-2-phenylindole (DAPI, 1 : 1000) and Phalloidin-AlexaFluor 488® (1 : 50). A Zeiss LSM 800 Airyscan confocal microscopy, with 405 nm, 488 nm and 640 nm diode lasers and 63× oil objective, was used to obtain the images. A step size of 0.34 μm was employed when acquiring Z-stack images.

## Conflicts of interest

There are no conflicts to declare.

## Supplementary Material

RA-014-D3RA08762J-s001
