# Supplementary material for: Modification of nanodiamonds for fluorescence bioimaging
Source: RSC Adv. 2024 Feb 5;14(7):4633–44. doi: 10.1039/d3ra08762j (PMC10839752; doi:10.1039/d3ra08762j)
Supplement: RA-014-D3RA08762J-s001 [file RA-014-D3RA08762J-s001.pdf]

Electronic Supplementary Material (ESI) for RSC Advances.
This journal is © The Royal Society of Chemistry 2024

Modification of nanodiamonds for fluorescence bioimaging

Claudia Fryer,[a,b] Patricia Murray,[b*] Haifei Zhang[a*]

a Department of Chemistry, University of Liverpool, Liverpool, L69 7ZD

b Department of Women's and Children's Health, Institute of Life Course and Medical Sciences, University of Liverpool, Liverpool, L69 3GE

* Corresponding authors: p.a.murray@liverpool.ac.uk, zhanghf@liverpool.ac.uk

Supporting Information

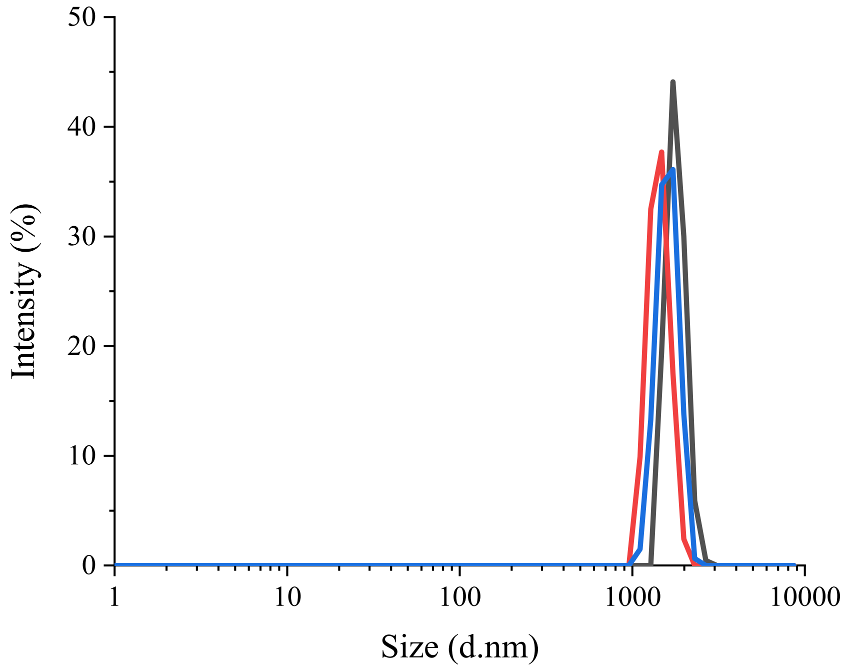

Fig. S1 Volume particle size distribution of detonation PDI-modified detonation NDs (0.1 mg/ml). The different colours represent 3 measurement scans with DLS.

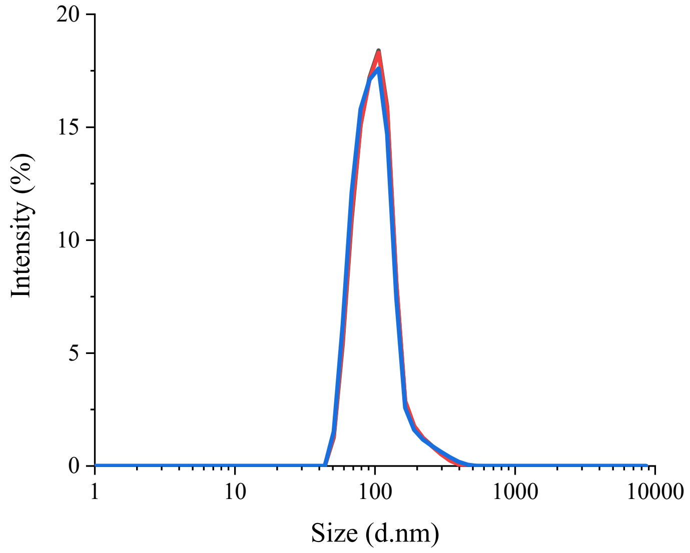

Fig. S2 Volume particle size distribution of PDI-modified HPHT NDs (0.1 mg/ml). The different colours represent 3 measurement scans with DLS.

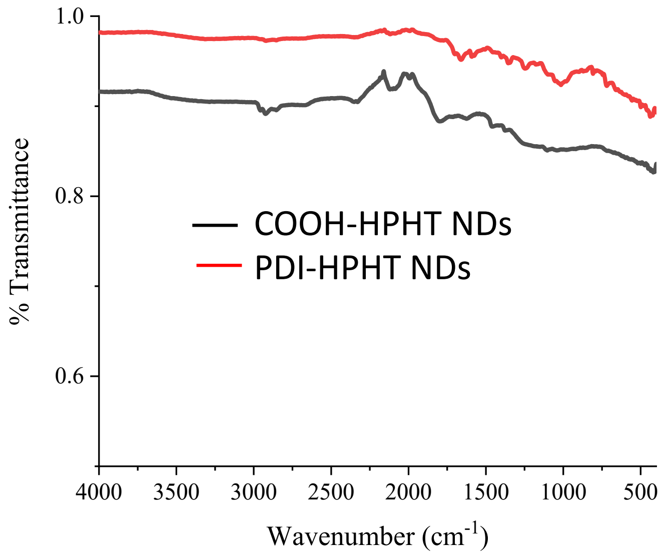

Fig. S3 FTIR spectra of COOH-HPHT NDs and after its functionalization with PDI.

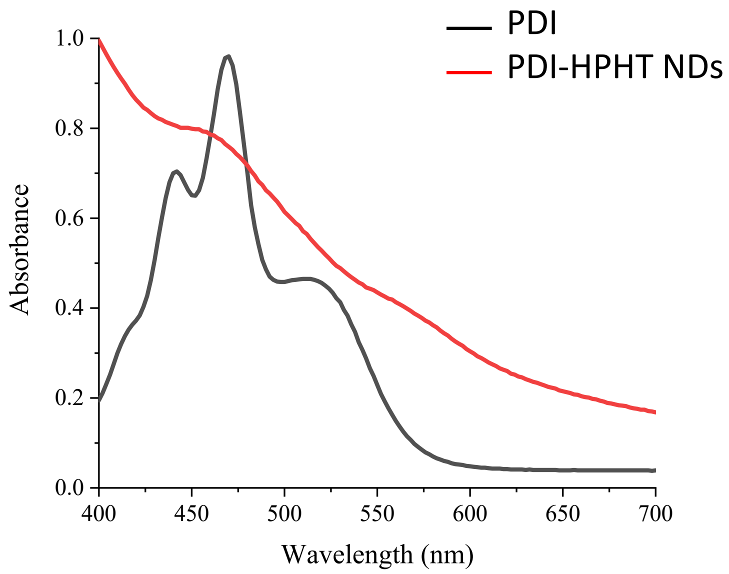

Fig. S4 UV-vis spectra of PDI-HPHT NDs (water, ~0.1 mg/ml) and PDI (0.06 mM NaOH solution).

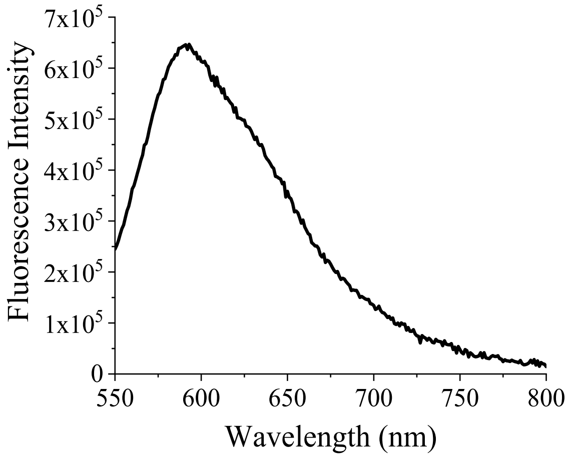

Fig. S5 Emission spectra (λ_{ex} = 500 nm) of pure PDI (0.06 mM NaOH solution).

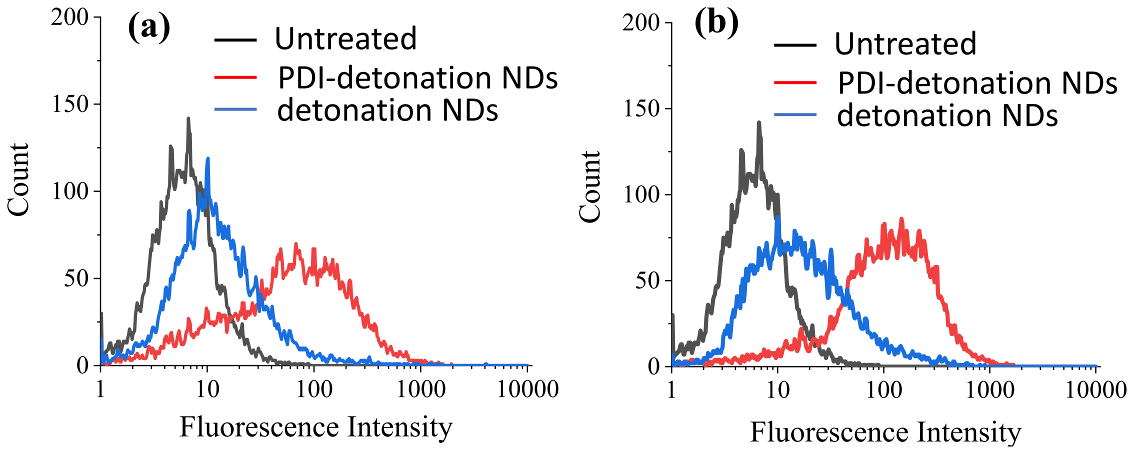

Fig. S6 Fluorescence intensity of MSCs labelled with both PDI-detonation NDs and detonation NDs at the concentration of (a) 25 μg/ml and (b) 100 μg/ml. Fluorescence intensity was measured using FL3 filter (670 LP), where 10,000 events were counted.

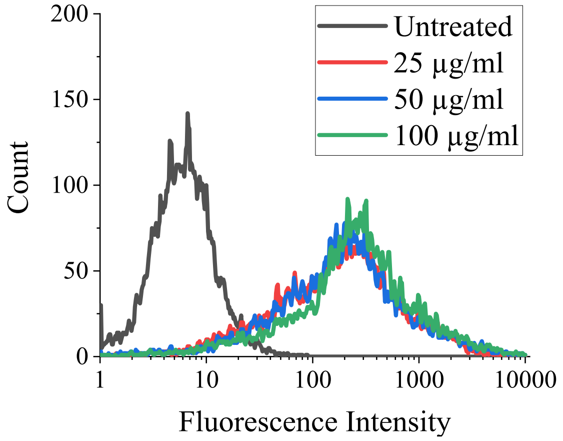

Fig. S7 Live cell flow cytometry of MSCs in PBS untreated and labelled with PDI-HPHT NDs at different concentrations. Fluorescence intensity was measured using FL3 filter (670 LP), where 10,000 events were counted.

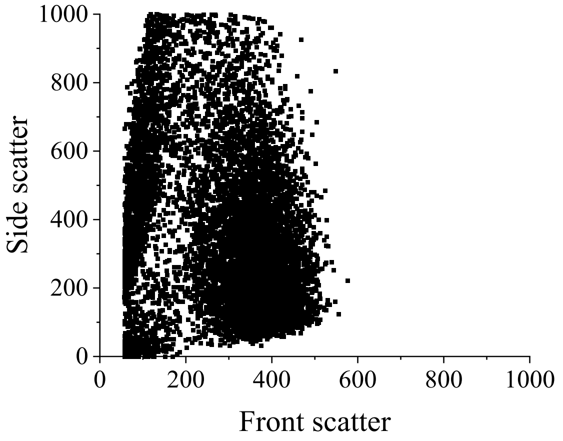

Fig. S8 Live cell flow cytometry of MSCs in PBS treated with PDI-modified HPHT NDs. The dot plot of side scatter vs. front scatter (25 μg/ml) is shown here.
